# KARGAMobile: Android app for portable, real-time, easily interpretable analysis of antibiotic resistance genes *via* nanopore sequencing

**DOI:** 10.3389/fbioe.2022.1016408

**Published:** 2022-10-17

**Authors:** Alexander Barquero, Simone Marini, Christina Boucher, Jaime Ruiz, Mattia Prosperi

**Affiliations:** ^1^ Department of Computer Science and Information and Engineering, University of Florida, Gainesville, FL, United States; ^2^ Department of Epidemiology, University of Florida, Gainesville, FL, United States; ^3^ Department of Pathology, University of Florida, Gainesville, FL, United States

**Keywords:** bioinformatics and computational biology, sequencing analyses, mobile apps, metagenomics, bacterial genes, antimicrobial resistance

## Abstract

Nanopore technology enables portable, real-time sequencing of microbial populations from clinical and ecological samples. An emerging healthcare application for Nanopore includes point-of-care, timely identification of antibiotic resistance genes (ARGs) to help developing targeted treatments of bacterial infections, and monitoring resistant outbreaks in the environment. While several computational tools exist for classifying ARGs from sequencing data, to date (2022) none have been developed for mobile devices. We present here KARGAMobile, a mobile app for portable, real-time, easily interpretable analysis of ARGs from Nanopore sequencing. KARGAMobile is the porting of an existing ARG identification tool named KARGA; it retains the same algorithmic structure, but it is optimized for mobile devices. Specifically, KARGAMobile employs a compressed ARG reference database and different internal data structures to save RAM usage. The KARGAMobile app features a friendly graphical user interface that guides through file browsing, loading, parameter setup, and process execution. More importantly, the output files are post-processed to create visual, printable and shareable reports, aiding users to interpret the ARG findings. The difference in classification performance between KARGAMobile and KARGA is minimal (96.2% *vs*. 96.9% f-measure on semi-synthetic datasets of 1 million reads with known resistance ground truth). Using real Nanopore experiments, KARGAMobile processes on average 1 GB data every 23–48 min (targeted sequencing - metagenomics), with peak RAM usage below 500MB, independently from input file sizes, and an average temperature of 49°C after 1 h of continuous data processing. KARGAMobile is written in Java and is available at https://github.com/Ruiz-HCI-Lab/KargaMobile under the MIT license.

## Introduction

Advances in high-throughput sequencing technologies have brought miniaturization and increased speed output of devices, permitting to perform experiments *in situ* and in real-time (Runtuwene et al., 2019). Oxford Nanopore’s MinION is the smallest sequencing device available in the market, measuring 10.5 × 2.3 × 3.3 cm, weighing 87 g, and USB powered. The MinION output is 420 nucleotide bases per second, with a maximal sequence read of 4 Megabases, and a maximal yield of 50 Gigabases over a 72-h run (https://nanoporetech.com/products/minion). The MinION can be used for both targeted whole genome sequencing (Loman et al., 2015) and metagenomics (Nicholls et al., 2019).

An emerging healthcare application for Nanopore includes point-of-care, timely identification of antibiotic resistance genes (ARGs) to help tailoring treatment in bacterial infections and monitoring outbreaks in the environment (Peter et al., 2020). Antimicrobial resistance (AMR) occurs when an organism acquires resistance to one or more antimicrobials (e.g., antibiotics), making it more challenging to treat and prevent spread across individuals and environments. AMR is a global public health and ecological concern with high mortality and economic costs worldwide (Prestinaci et al., 2015; Dhingra et al., 2020) and it was classified by the World Health Organization (WHO) as one of the “top ten global health threats” in 2019. Close to three million resistant infections occur in the United States each year and more than 35,000 people die as a result (CDC, 2019). Frequently, the goal is either testing a clinical sample to determine its resistance to various antibiotic treatments, or monitoring an environment on a routine basis to determine public health risks such as in foodborne infections. The traditional form of testing for AMR is *via* sample culture and vitro phenotypic antibiotic susceptibility testing (Vasala et al., 2020). Since the majority of microbial species is unculturable and cannot live outside their natural environment, phenotypic resistance testing is inadequate in a large number of settings. However, sequencing technologies have become more readily available and their application for AMR surveillance is more widespread. Here, we note that AMR is largely determined by the ARGs found in the DNA of a biological sample, making the use of genome sequencing (both affordable and fast) to characterize resistance feasible; one only needs to have an accurate computational tool to identify ARGs (Hendriksen et al., 2019; Lv et al., 2021). Several online resources for ARGs as well as genotype-phenotype data are available, including the Pathosystems Resource Integration Center (PATRIC), the Comprehensive Antibiotic Resistance Database (CARD), and MEGARes (Alcock et al., 2019; Davis et al., 2019; Doster et al., 2019). In parallel, several computational tools for characterization of ARGs from sequencing data (both whole genome and metagenomics) exist, including AMRPlusPlus (Doster et al., 2019), DeepARG (Arango-Argoty et al., 2018), KARGA (Prosperi and Marini, 2021), MetaMARC (Lakin et al., 2019), Resfinder (Bortolaia et al., 2020), AMR-meta (Marini et al., 2022b), and VAMPr (Kim et al., 2020). Some of these tools work directly on short read data, while others on assembled contigs or draft genomes. Overall, most of them require a significant amount of memory and computational power. A comprehensive benchmark on clinical samples has been published by Marini et al. (Marini et al., 2022a).

Surveillance of AMR is particularly important in rural areas, where there is a significant amount of antimicrobial use. In fact, over 80% of antibiotics usage in the United States is relative to food production animals (swine, cattle, and poultry). This has been cited as the cause for increased drug-resistant infections in areas with abundant farming (Manyi-Loh et al., 2018). Unfortunately, rural areas (e.g., farms, food production facilities and clinics) frequently lack the sequencing facilities and computational resources that have been used to generate and analyze shotgun genomics data for AMR detection. However, third-generation sequencing technologies have enabled portable sequencing and remove the laboratory burden. These miniaturized, battery-powered sequencers take as input a biological sample and produce high throughput sequencing data that is transferred on a portable device, such as a smartphone (Check Hayden, 2015). There is now availability of portable kits that allow both the sample preparation and sequencing to be done on-site within 30 min, e.g., the VolTRAX (https://nanoporetech.com/products/voltrax).

The challenge that remains is a computational one, i.e., the sequencing technology is now portable but the bioinformatics analysis is not, defying the purpose of the portability itself. The data is required to be transferred from the portable device to a high-performance server or computing cloud in order to perform the analysis. The Nanopore MinION must be connected to a desktop that is powerful enough to perform data analyses, or–more likely–to transfer the data elsewhere for analyses. Both commercial software for Nanopore analytics (e.g., Metrichor, https://metrichor.com/) and open-source tools (e.g., Poretools (Loman and Quinlan, 2014), PoreSeq (Szalay and Golovchenko, 2015), poRe (Watson et al., 2015), Nanocall (David et al., 2017), Minimap2 (Li, 2021)) require the transfer of data to hardware with specific confiugurations and capabilities (e.g., Linux/UNIX, SIMD-SSE acceleration, software library dependencies).

One of the challenges of large data transfers is that many of the rural communities do not have high-speed broadband deployed (Kang, 2019; Kennedy, 2019). According to the US Federal Communications Commission (FCC)’s Eight Broadband Progress Report, 19 million Americans still lack access to broadband service at threshold speeds. In rural areas, nearly one-fourth of the population lack access to this service and in tribal areas, nearly one-third of the population lacks access. Thus, although the necessary data can be generated in resource-limited areas *via* third generation sequencing, they cannot be analyzed on-site. From an epidemiology and public health perspective, this delays bacterial outbreak surveillance and quantification of AMR in critical areas where even healthcare services might be delayed. Furthermore, the transport of a powerful desktop/laptop computer may present some disadvantages in mobile labs. Two reasons are weight and sterilization. If researchers have to carry their equipment for several miles, even just a laptop adds 2–5 Kg in weight and volume, without considering the power needs A “daysack-scale” portable shotgun sequencing kit has been estimated to weigh ∼10 kg, including a 12-V DC micro centrifuge, a Nanopore sequencer and flow cell, a ruggedized laptop, and a multi-voltage power pack, among the other items (Edwards et al., 2022). A tablet or a phone both have minimal weight, volume, require less power, and can be charged with portable batteries. For sterilization purposes, tablets and mobile phone are also much easier to deal with, as they are generally water-resistant, thus can be sprayed and wiped with antiseptic solution. While it is true that a laptop is needed in several steps of the Nanopore sequencing, there are also bundled devices that relax such requirement; for instance, the MinION Mk1C (450 g weight, 14 cm × 3 cm size) comes with pre-installed basecalling and analysis software. In a prior work, we also showed that basecalling can be performed on smartphones (Oliva et al., 2020).

To date (2022), there is no ARG detection software available that runs on mobile devices. One of the reasons is that the software needs to be recompiled for mobile chipsets, and this is not always feasible due to lack of libraries or instruction sets (Oliva et al., 2020). In fact, code often needs to be re-implemented (Palatnick et al., 2020) in a manner that it accounts for RAM constraints and device overheating (Milicchio and Prosperi, 2021).

In this paper, we present KARGAMobile, a mobile app for portable, real-time, easily interpretable analysis of ARGs from Nanopore sequencing data. It does not require any transfer of data, eliminating the need for a high-speed internet connection. All computations are done within the mobile device hardware, without external CPUs. The code is written entirely in Java, without any external dependency, and it works within the memory constraints of any off-the-shelf Android OS. Our app ports the code–and optimizes it for mobile hardware–of an existing, validated algorithm called KARGA. We show that the ARG detection performance of KARGAMobile is in line the original KARGA and with other AMR classification tools. KARGAMobile features a graphical user interface and generates visual summary reports, shareable and exportable. Speed of execution on real datasets from hospital outbreaks demonstrates its applicability in real-time scenarios. Thus, KARGAMObile effectively enables detection of AMR in rural environments that are resource-limited and we expect a beneficial impact for public health.

## Methods

### KARGAMobile algorithm

KARGAMobile’s algorithm is derived from an extensively validated ARG classification method named KARGA (Prosperi and Marini, 2021; Marini et al., 2022a). KARGA classifies a DNA sequence–in the form of sequence read from a FASTQ file–as part of an ARG (or not) by employing a statistical approach that compares the *k*-mer spectrum of the read with that of all ARGs obtained from a given database (including reverse complements). Here, we define a *k*-mer of a sequence (or of a collection of sequences) as a substring made of *k* consecutive characters. The *k*-mer spectrum is defined as the set of all the *k*-mers of a sequence (or of a collection of sequences) along with their frequencies (because a *k*-mer can be found in multiple positions of a sequence set). KARGA’s reference ARG database is MEGARes, which is selected due to its comprehensiveness and well-structured AMR ontology, comprised of a hierarchical, multi-level structure, going from AMR type, to class, to mechanism, to group (Doster et al., 2019). Of note, KARGA does not include genes that are responsible for antibiotic resistance through point mutations, which are called ARG variants (ARGVs) (Woodford and Ellington, 2007); this is because the current version of MEGARes (2.0) flags ARGVs, but does not provide confirmation of mutations’ presence.

**FIGURE 1:**
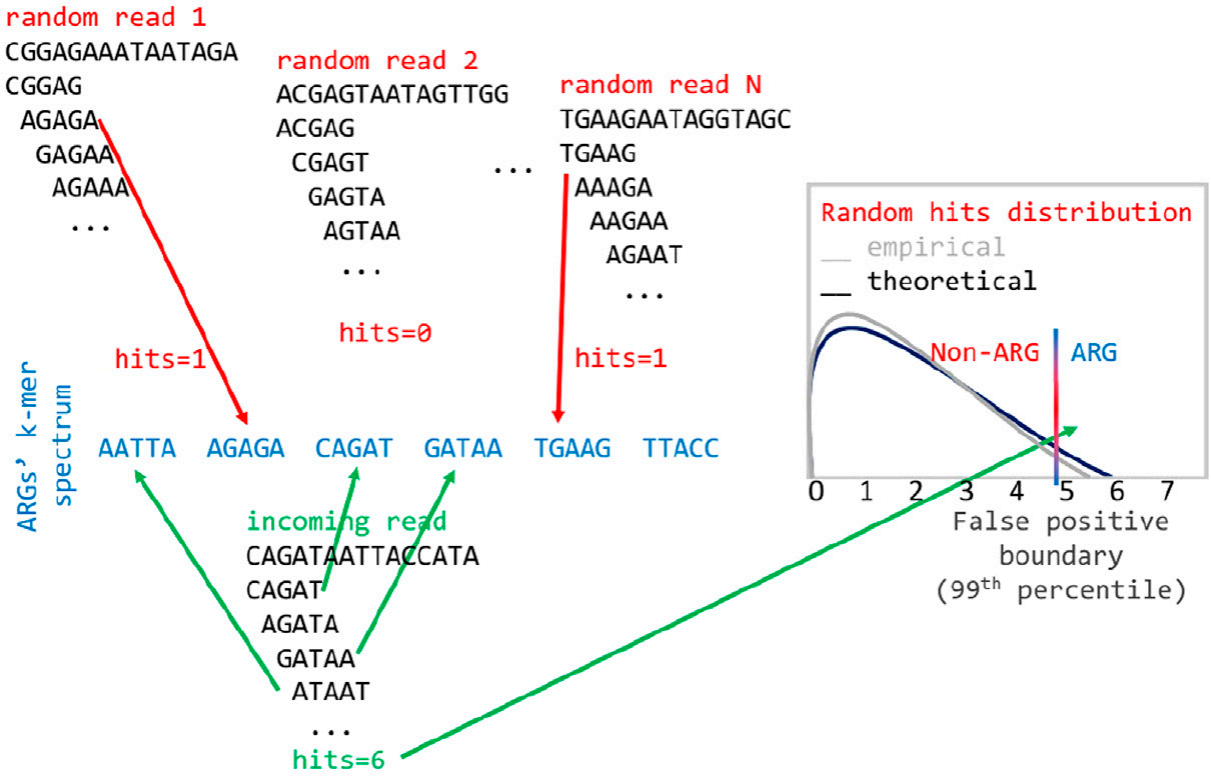
Empirical distribution of matches of random *k*-mers to the ARG spectrum to estimate the desired threshold of false positive rate.

**FIGURE 2:**
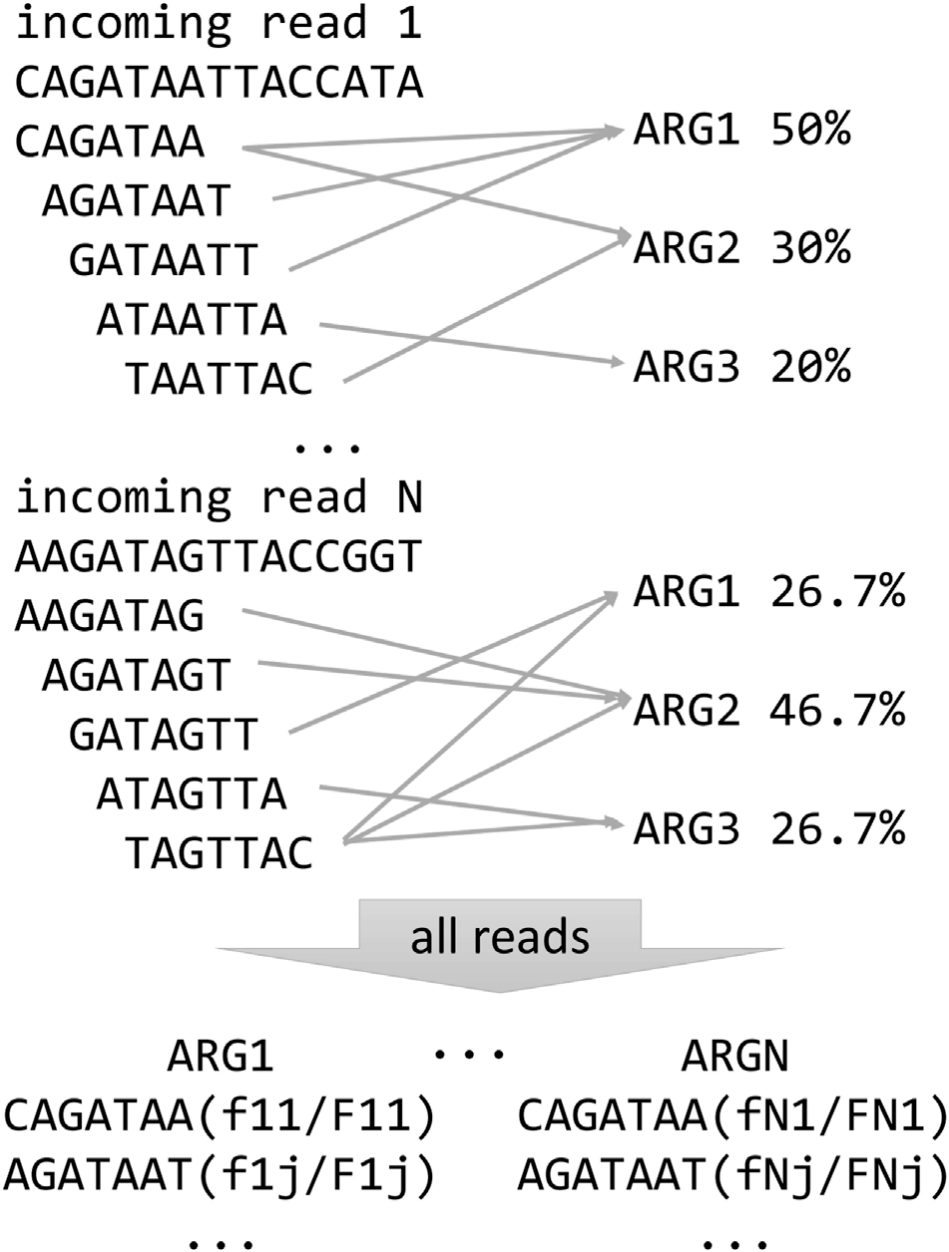
Schematic representation of KARGA’s ARG classification algorithm for individual reads and resistome summary for the whole sample.

When classifying a read, the algorithm first uses a statistical test apt to minimize the probability of a false positive match with the ARG database. Thus, it is possible that no ARGs are reported for one read if the test fails. Specifically, for each read, the statistical test verifies that the number of *k*-mers matching the ARG *k*-mer spectrum is higher than the expected number of matches from a null distribution of non-ARG *k*-mer matches, for given false positive rate. The false positive distribution is calculated by matching *k*-mers of random reads (with the same average length and standard deviation as the real data) to the ARG spectrum. We employ an empirical calculation of the distribution based on random read simulation, which is very close to the theoretical estimate, with the advantage of being very easy to implement (Prosperi et al., 2012). Here, we set the false positive rate to 0.01, and we discard all reads whose number of *k*-mer matches with the ARG spectrum is below the number of matches from the null distribution corresponding to the 99th percentile (Figure 1). If the read passes the test, based on the spectrum comparisons, the algorithm calculates the probability that the read comes from one or more ARGs, using a multinomial classification. In detail, when a *k*-mer from a read matches to more than one ARG, it is assigned a fractional score; for instance, if it matches to 5 genes, the score is 0.2. Then, all the scores from all *k*-mers of a read are summed up for each ARG and normalized by the total counts. In this way, a vector of probabilities that sums up to 1, i.e., a multinomial distribution, is created for each read (Figure 2). KARGA has two modalities for classifying reads: ‘best-match’ and ‘multinomial.’ The best-match reports the most probable ARG (if the statistical test is passed), while the multinomial reports all matching ARGs, ranked by decreasing probability, up to 95% cumulative. By default, reads are classified at the most detailed group level (according to MEGARes ontology). However, it is possible to classify at higher levels, e.g., antibiotic mechanism or class, using the information provided in the output from single or multiple read classifications.

In addition, after all input sequences are processed, KARGA creates a file with a description of the overall resistome of the sample. In other words, for each ARG detected in the reference database, KARGA prints its sample coverage and depth (Figure 2). The coverage of an ARG is defined as the number of *k*-mers matched by at least one read (in forward, reverse strand, and counting its repetitions in the ARG), while the depth is how many times on average an ARG *k*-mer was matched by considering all reads. Note that when the multiple multinomial classification is enabled, the resistome output can also change, since a read can be assigned to more than one gene (in a weighted way).

KARGA makes use of a double-lookup strategy that links *k*-mers to ARGs and ARGs to *k*-mers, implemented as two-level nested hash tables (HashMap < String, HashMap < String, Integer ≫) to assure efficient querying time. All *k*-mers are also stored in both forward and reverse strand to avoid the need of reversing each single input read. Due to limitations of Android development, we are required to limit the memory usage to 512 MB RAM. To accomplish this, we re-implemented KARGA in the following ways: 1) we replaced one of the two-level nested hash tables with an array structure with Integer indexing of ARGs (HashMap < String, ArrayList < Integer ≫), which is the level with the lowest expected number of queries; 2) we removed all reverse complements *k*-mers from the hash tables, calculating instead the reverse complements of reads on the fly during processing; and 3) lastly, we created a compressed version of MEGARes by reducing redundancy in the ARGs and retaining only one gene representative among those that cluster together at very high similarity. Specifically, we used CD-HIT (Li and Godzik, 2006) with a threshold of 92.5%. With these changes, the KARGAMobile implementation runs within 512 MB of RAM within a wide range of operating, realistic parameter values (i.e., *k* between 13 and 45) and the RAM usage remains constant regardless input file size (FASTQ or gzipped FASTQ) or read size (i.e., between 150 and 10,000 bases).

One thing to note is that KARGAMobile uses an in-memory database which is loaded at each program execution, without any data transforms or succinct structures, e.g., integer hashing of strings, Burrows-Wheeler transform, or FM-index (Shibuya, 2019). ARGs are several orders of magnitude smaller in size than bacterial genes and genomes. The largest ARG database available to date (2022) is MEGARes, which contains about 8,000 gene entries for a total of 3.5 Mb. While it is true that bacterial genome collections increase at a high pace, the same is not true for ARGs. This is due to the fact that many genes are shared across species, so discovery of new species does not necessarily translate into discovery of new ARGs. Also, new ARGs require laboratory confirmation of AMR (Hu et al., 2016; Evans et al., 2020), so the confirmation process takes longer and it is bound by the current drugs available. More drugs could lead to new ARGs, but the time scale for the development and introduction of a new drug is in terms of years. Thus, while the in-memory, standard data structure choice might not be elegant or scalable, it favors code simplicity and does not require any external dependency. KARGAMobile will work with the factory Java virtual machine on Android OS.

### Design of the mobile application

KARGAMobile, the smartphone application built on the Java code of KARGA, is developed entirely in the Android Studio Integrated Development Environment (IDE) version 2021.2.1 (https://developer.android.com/studio/releases). The target Application Program Interface (API) level for the application is API level 31, although it supports down to a minimum API level 28. Android Studio suggests approximately 69% of all Android devices will support applications developed in API level 28. By targeting the API level 31, the application is intended to be as time resilient as possible, being that Google Play’s policies require for all apps to target this level or above starting November 2022. KARGAMobile also takes advantage of both novel and well-known Android app interface design components, such as the Android Jetpack (https://developer.android.com/jetpack) and the MPAndroidChart (https://github.com/PhilJay/MPAndroidChart) libraries. These libraries are used to create some of the different interface objects that allow the app to achieve its purpose.

#### App interface

**FIGURE 3:**
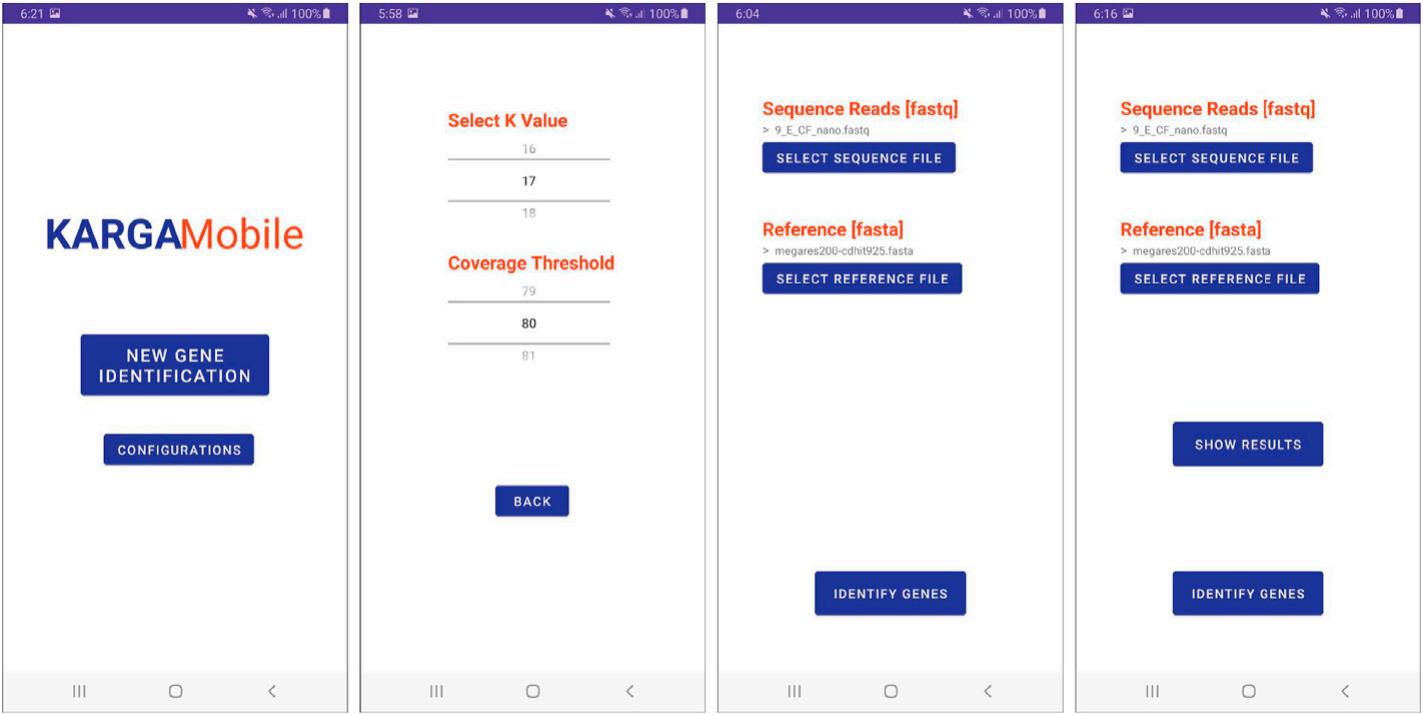
Graphical user interface of the KARGAMobile app. The figure shows (left to right) the start menu, configuration, file selection and run screens.

The app interface was created by following a simplified version of the user-centered design process (Norman and Draper, 1986). To design the interface, we used multiple user interface and user experience (UI/UX) design artifacts, including paper prototypes and wireframes, with strong involvement of the original KARGA’s developers as well as potential end users (Jacko, 2012). Other decisions regarding UI/UX, such as objects location, fonts, and color selection, were made following best practices, Android recommendations for developers and the gestalt principles of design (Rogers et al., 2011). In general terms, these and all the other different interface components were created to better support the KARGA tool functionality in a smartphone graphical user interface (GUI) context and environment that is naturally different from the original command-line interface, with no GUI. Thus, the application was developed to include interface features that support and extend all of the available functions. When the user starts the application for the first time, they are asked to enable KARGAMobile to access the phone’s memory and storage. This is required to enable the app to load the sequence and reference files and to store all the different results. The user is then presented with the home menu shown in Figure 3, where they can navigate to the main features of the application. Touching the *‘Configurations’* button opens the screen that allows the user to change application-wide configuration values required for ARG analysis, as seen in the second image in Figure 3. The current version of KARGAMobile allows the user to set the *k* parameter (from 13 to 45, with a default value of 17), as well as the percentage coverage threshold (from 0% to 100%, with a default value of 80%) of the ARGs that will be displayed in the results. In the home menu, the user can also select the *‘New Gene Identification’* option, which opens the main function screen shown in the third image from the left in Figure 3. In this screen, the user is prompted to choose both the input read file and the reference database that will be used as parameters for the ARG analysis. While there is virtually no limit to the read file size (except for longer processing times and potential device overheating), the database file must be calibrated to meet the RAM requirements. Once any of the select file buttons have been pressed, Android’s own file picker system is invoked by KARGAMobile, which serves the purpose of providing the user with a file selection solution that should feel familiar and safe to them. Thanks to the permissions that were granted the first time, these files can be located anywhere in the user’s phone memory or in any attached microSD card. When both files have been selected, the system enables the ‘Identify Genes’ button, which once pressed, starts and runs through the whole ARG analysis process. Since Android’s regular worker threads are constrained to a ten minute limit (and the ARG analysis can run for longer times), we used high-priority foreground asynchronous threads with long-running capabilities (https://developer.android.com/topic/libraries/architecture/workmanager/advanced/long-running) and implemented the mechanisms to communicate back with our app and be notified once the analysis process was completed.

#### Visualizing results

**FIGURE 4:**
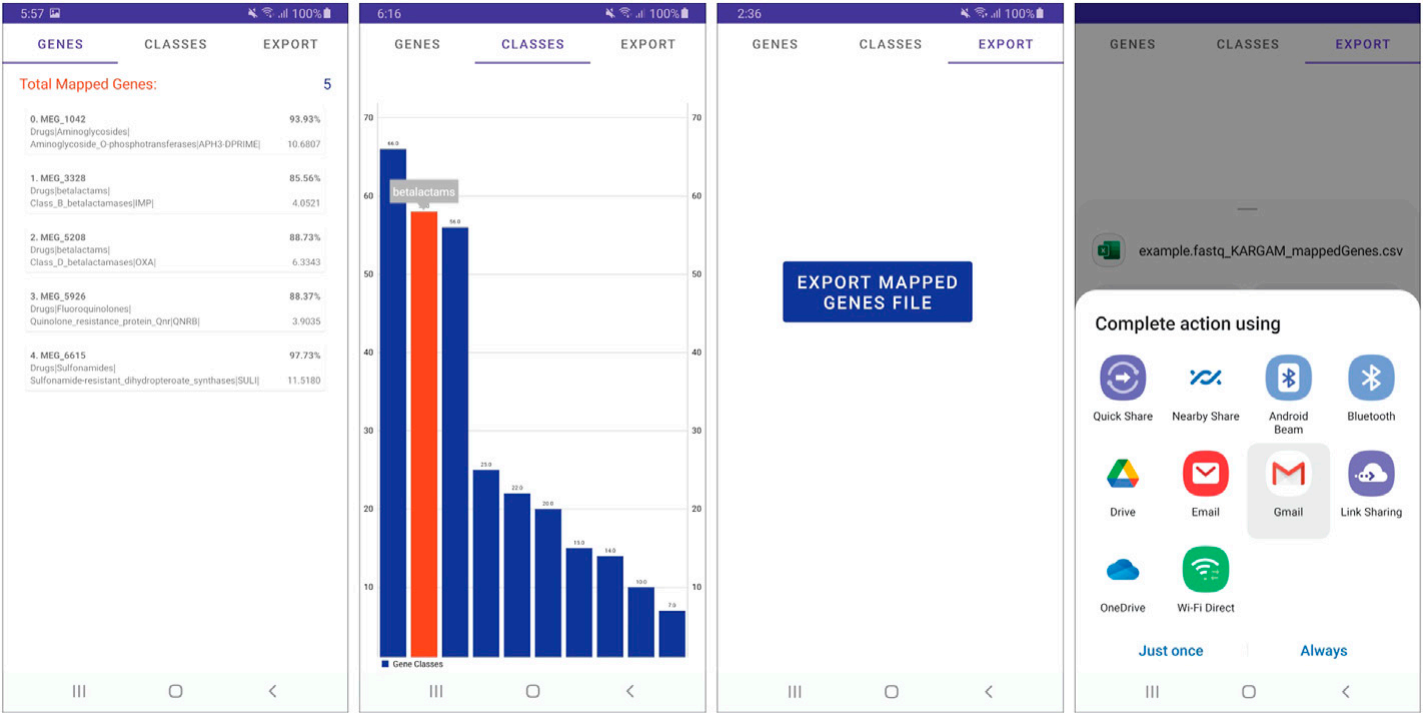
Screenshots of the KARGAMobile app after completing a run, showing (from left to right) the ARG coverage and depth summaries in textual mode, the visual charts of ARGs grouped by AMR classes, and the export/sharing data options.

When the ARG analysis is done, the app enables the ‘Show Results’ button that can be seen in the rightmost screen in Figure 3. It is also at this point that the system automatically creates and stores a comma-separated value file with every identified ARG in the default app location folder. This file can be kept for future reference and can also be sent to others. When the ‘Show Results’ button is pressed, the user is taken to the results screen, which can be seen in the first image in Figure 4. The results screen tab menu give the user three different options: “Genes”, “Classes”, and “Export’, with the “Genes” tab selected by default. In this first tab, the user is presented with the resulting ARG list. To display the ARG list directly in screen, the app makes use of a memory efficient solution provided by Android Jetpack’s RecyclerView (https://developer.android.com/guide/topics/ui/layout/recyclerview). With this component, the system is able to dynamically display large lists of items that only consume phone resources when the objects are being rendered on screen. Each item that comes into view is then displayed by automatically parsing all the ARG information into another Android component, called a CardView object. Using CardView works as a convenient graphical template that maintains a normalized visual aesthetic, which can also be easily modified in future app versions. In our tests, we were able to render ARG lists with approximately 5,000 different objects without delays when scrolling through the item collection. When the user taps the second tab, “Classes”, the app displays a bar graph that represents the top 10 most frequent ARG classes in the list, as seen in the second image of Figure 4. This graph can be easily explored and manipulated using common touch gestures such as the two-finger pinch, which allows the user to zoom-in and out on their element of choice. Also when selected, each bar in the graph will be highlighted in a different color and indicate the ARG class that it represents. Finally, the last tab in the Results section, ‘Export’, provides the user with the sharing functionality, which in turn leverages underlying Android capabilities (https://developer.android.com/training/secure-file-sharing/share-file), as seen on the last two images in Figure 4. With this feature, the user can send the ARG list to any of the different apps in their phone, be it messaging, e-mail, collaboration and work, or cloud-storage.

### Data and experimental settings

To validate KARGAMobile’s performance in detecting ARGs, we used first semi-synthetic datasets and then real, experimental Nanopore data. The semi-synthetic data that included ARGs from the MEGARes database v.2.0 (ground truth for antibiotic resistance) and genes sampled from a vertebrate genome (ground truth for non-ARG genes), the *Sus scrofa*. MEGARes contains about 8,000 ARGs responsible for antibiotic resistance (with the top-5 being betalactams, aminoglycosides, glycopeptides, fluoroquinoloes, and tetracyclines), as well as metals and biocides. The datasets were generated using PBSIM2 (Ono et al., 2020), with Nanopore-specific chemistry simulation (ratio of differences for substitutions, insertions, and deletions set as 23, 31, and 46), tailored for bacteria and vertebrate organisms (PSBIM2 models R10.3 and R9.4, respectively). Target coverage was 10x, 32x, and 64x for MEGARes, and 0.5x, 1x, and 2x for *Sus scrofa*. According to coverage, three datasets were generated, made of 165,038 (165K), 537,927 (500K), and 1,070,717 (1M) reads. The model-based median (min-max) error rate was set to the default value for Nanopore, which was 15% (0%–35%), and all other PBSIM2 parameters were also set to Nanopore defaults. We calculated overall accuracy and false positive rate, comparing results against KARGA. Also, the parameter *k* was optimized to maximize performance with Nanopore data, since the default value of KARGA is tailored to Illumina sequencing technology.

The real Nanopore experiments included both targeted whole genome sequencing and metagenomics. The targeted sequencing data were previously published by (Peter et al., 2020), who tracked ARGs in outbreaks of *Citrobacter cronae*, *Citrobacter freundii*, and *Pseudomonas aeruginosa* from a German hospital over six years. The metagenomics data have been presented by (Yang et al., 2019), who analyzed clinical respiratory specimens from people hospitalized with severe pneumonia (both culture-positive and culture-negative) undergoing mechanically-ventilation. These Nanopore data are available publicly at https://www.ncbi.nlm.nih.gov/sra/?term=PRJEB31907 and https://www.ncbi.nlm.nih.gov/sra/?term=PRJNA554461, respectively.

All tests were performed on a Samsung Galaxy S9+ smartphone (SM-G9650), with a Qualcomm Snapdragon 845 CPU, 6GB RAM, mounting Android OS 10. Wall/CPU time, memory profiling and temperature were measured directly in the app using Android’s available functionalities. All the different analytic tests run on a different thread that was constantly profiling the mapping thread and recording the values, generating a separate comma-separated value file with all the data at the end of every ARG analysis for future reference. This feature is also included as part of the KARGAMobile app and can be easily enabled or disabled through a global trigger directly in the code.

## Results

The KARGAMobile main menu screen allows the user to start a “New gene identification” analysis with standard parameters, or to open the “Configurations” menu to choose the parameters, as described in the Methods section. The main menu and configuration screenshots are shown in Figure 3, along with the file selection screen to set the ARG reference database (FASTA format, including the pre-loaded default) and the input read file (FASTQ or FASTQ.gz compressed format). After the analysis is completed, the user can visualize results in textual or graphical mode, as illustrated in Figure 4. The text mode summarizes ARGs by name, AMR ontology term (namely, antibiotic class, group and mechanism), coverage and depth. The graphical mode displays frequency bar charts aggregated at the AMR class level. All results can be shared and exported in different ways, including e-mail and cloud storage, or sent to a printer device.

The compressed version of MEGARes v.2.0 used in KARGAMobile included 2,050 ARGs, reduced from the original set of 7,378 (we here excluded chromosomal genes with mutations). The average (st.dev.) read length of the semi-synthetic validation datasets was 876 (±437) and 8,984 (±6,977) bases for MEGARes and *Sus scrofa*, respectively. Accuracy and false positive rate of the two tools were very similar, as shown in Table 1. From the validation results, the optimal value of *k* was 25 for both KARGAMobile and KARGA: on the largest dataset, the *f*_1_-measure was 96.9% for KARGA and 96.2% for KARGAMobile, the balanced accuracy was 97.3% and 96.9%, the false negative rate was 5.4% and 6.3%, with zero false positive rate. Of note, the *k* value is higher than the usual KARGA (non-mobile version) default. Two contrasting factors influence the optimal value: the read length drives the value up, with Nanopore reads longer than Illumina ones; and the error rate drives the value down, with Nanopore reads being more erroneous than Illumina ones. In fact, when looking at the resistome summary for the semi-synthetic data, values of *k* between 17 and 25 behaved better than 25, because they allowed to identify more genes at higher coverage, with the same false positive rate. Specifically, at *k* = 17, 100% of ARGs from MEGARes could be retrieved at least 50% coverage, with no spurious attributions of reads generated from the *Sus scrofa* genome. Conversely, at *k* = 25, only 89% of ARGs could be identified at 50% coverage, without any spurious *Sus scrofa* read assignment.

**TABLE 1:** Validation performance of KARGAMobile and KARGA on semi-synthetic datasets.

Method	set	*k*	FPR	FNR	BA	F1
KARGA	165K	13	49.7% (±0.2%)	4% (±0.1%)	73.2% (±0.1%)	78.1% (±0.1%)
	165K	17	19% (±0.1%)	1.4% (±0%)	89.8% (±0.1%)	90.6% (±0.1%)
	165K	25	0% (±0%)	6.3% (±0.1%)	96.9% (±0%)	96.8% (±0%)
	165K	31	0% (±0%)	12.8% (±0.1%)	93.6% (±0.1%)	93.2% (±0.1%)
	165K	45	0% (±0%)	43.9% (±0.2%)	78.1% (±0.1%)	71.9% (±0.1%)
KARGAMobile	165K	13	49.9% (±0.2%)	2.6% (±0.1%)	73.7% (±0.1%)	78.8% (±0.1%)
	165K	17	12.3% (±0.1%)	1.6% (±0%)	93.1% (±0.1%)	93.4% (±0.1%)
	165K	25	0% (±0%)	7.5% (±0.1%)	96.2% (±0%)	96.1% (±0%)
	165K	31	0% (±0%)	15.9% (±0.1%)	92.1% (±0.1%)	91.4% (±0.1%)
	165K	45	0% (±0%)	51.6% (±0.2%)	74.2% (±0.1%)	65.2% (±0.1%)
KARGA	500K	13	49.2% (±0%)	7.5% (±0%)	71.6% (±0%)	77% (±0%)
	500K	17	19% (±0%)	1.7% (±0%)	89.7% (±0%)	90% (±0%)
	500K	25	0% (±0%)	5.6% (±0%)	97.2% (±0%)	96.7% (±0%)
	500K	31	0% (±0%)	10.7% (±0%)	94.6% (±0%)	93.1% (±0%)
	500K	45	0% (±0%)	28.8% (±0%)	85.6% (±0%)	72.3% (±0%)
KARGAMobile	500K	13	49.6% (±0%)	5.1% (±0%)	72.6% (±0%)	77.7% (±0%)
	500K	17	12.1% (±0%)	1.7% (±0%)	93.1% (±0%)	93.1% (±0%)
	500K	25	0% (±0%)	6.6% (±0%)	96.7% (±0%)	96.1% (±0%)
	500K	31	0% (±0%)	1.3% (±0%)	93.5% (±0%)	91.3% (±0%)
	500K	45	0% (±0%)	3.2% (±0%)	83.9% (±0%)	65.8% (±0%)
KARGA	1M	13	49.2% (±0%)	6.3% (±0%)	72.2% (±0%)	77.2% (±0%)
	1M	17	1.8% (±0%)	1.6% (±0%)	89.8% (±0%)	90% (±0%)
	1M	25	0% (±0%)	5.3% (±0%)	97.3% (±0%)	96.9% (±0%)
	1M	31	0% (±0%)	10.4% (±0%)	94.8% (±0%)	93.3% (±0%)
	1M	45	0% (±0%)	28.4% (±0%)	85.8% (±0%)	72.6% (±0%)
KARGAMobile	1M	13	49.4% (±0%)	4.1% (±0%)	73.2% (±0%)	77.8% (±0%)
	1M	17	12.1% (±0%)	1.6% (±0%)	93.1 (±0%)	93.1% (±0%)
	1M	25	0% (±0%)	6.3% (±0%)	96.9% (±0%)	96.2 (±0%)
	1M	31	0% (±0%)	12.6% (±0%)	93.7% (±0%)	91.6% (±0%)
	1M	45	0% (±0%)	31.9% (±0%)	84% (±0%)	66.1% (±0%)

FPR: false positive rate; FNR: false negative rate; BA: balanced accuracy; F1: *f*_1_-measure.

We then tested KARGAMobile on the real Nanopore experimental datasets from targeted sequencing of hospital outbreaks, and metagenomics sequencing of mechanically-ventilated patients with severe pneumonia. Respectively, we used 14 FASTQ files from (Peter et al., 2020), ranging from 238MB to 5,779MB, and 6 FASTQ files from (Yang et al., 2019), ranging from 221MB to 775 MB. Table 2 gives details on run times, memory and temperature usage. On the targeted bacterial sequencing data, KARGAMobile processed on average 1 GB in 23 min, with a peak RAM usage of 498 MB independently from input file size and average/peak temperature of 49/60°C after 1 h of continuous data processing. In fact, when executing a formal one-sample *t*-test for possible deviations from the null hypothesis of independence of RAM usage and temperature, none of the experiments yielded a *p*-value lower than 0.64. On the metagenomics data, the processing speed was 48 min per 1GB, the average peak RAM usage was 394MB, and the average/peak temperature was 46/53°C after 30 min of continuous data processing.

**TABLE 2:** Run summary of KARGAMobile on real experimental data: (1) targeted sequencing of *C. cronae*, *C. freundii*, and *P. aeruginosa* in hospital outbreaks; (2) metagenomics experiments from mechanically-ventilated patients hospitalized with severe pneumonia.

	Accession	File size (MB)	CPU/wall time (mm)	Avg/max RAM (MB)	Avg/max temp. (°C)
Targeted sequencing	ERX3333096	228	5/5	323/476	44/49
	ERX3333087	538	12/12	323/506	47/52
	ERX3333084	810	23/24	321/512	47/58
	ERX3333097	877	22/23	320/512	50/55
	ERX3333088	925	23/24	321/512	48/53
	ERX3333095	1041	24/25	321/512	49/56
	ERX3333085	1088	22/23	317/459	46/52
	ERX3333086	1137	22/23	323/511	47/54
	ERX3333093	1468	34/35	320/512	49/55
	ERX3333090	3521	74/76	319/512	49/60
	ERX3333094	4424	96/99	322/512	49/58
	ERX3333092	4639	104/106	319/499	50/63
	ERX3333091	4931	105/108	319/473	49/60
	ERX3333089	5779	115/118	319/462	49/60
	Avg. (st.dev)	2243 (1943)	49 (40)/50 (41)	321 (2)/498 (21)	48 (2)/56 (4)
Metagenomics	SRX6447026	221	12/13	317/383	44/51
	SRX6447019	382	17/18	320/409	46/55
	SRX6446993	397	17/18	320/397	46/52
	SRX6447061	473	22/22	317/376	40/48
	SRX6447024	483	21/21	319/402	44/51
	SRX6447018	775	34/35	318/397	46/53
	Avg. (st.dev)	455 (183)	20 (7)/21 (7)	318 (1)/394 (12)	44 (3)/52 (2)

In order to better investigate the temperature usage and possible critical overheating, we run consecutive tests on a single device, using the metagenomics data, until the device shut off for overheating, or a five-hour limit was reached. After 5 h and 30 min of wall time (05:15 h h:mm of CPU time), 25 files had been completed successfully, using an average RAM of 318 MB (peak of 512 MB), at an average temperature of 44.6°C (max of 59.1°C). During the whole process, the device never shut off for overheating.

In Table 3, we show the distribution of ARG findings for both the targeted sequencing an the metagenomics datasets using gene coverage thresholds of 50% and 80%, counting the number of different ARGs and ARG classes. For both datasets, it is notable that while the ARG number increased sensibly by decreasing the ARG coverage threshold, the detection at the class level was less affected. More ARGs were detected in the targeted sequencing data because the biological samples had high yield of antimicrobial resistance findings, while some of the sample cultures from the respiratory samples used for metagenomics came culture-negative. Of note, even if the ARG summaries are normalized by the ARG database size, difference in species coverage does not allow a direct comparison between the two experimental setups.

**TABLE 3:** Distribution of ARG findings by KARGAMobile using gene coverage thresholds of 80% and 50%, counting the number of different ARGs and ARG classes, on real experimental data: (1) targeted sequencing of *C. cronae*, *C. freundii*, and *P. aeruginosa* in hospital outbreaks; (2) metagenomics experiments from mechanically-ventilated patients hospitalized with severe pneumonia.

	Accession	ARGs (80%)	Classes (80%)	ARGs (50%)	Classes (50%)
Targeted sequencing	ERX3333096	5	4	12	7
	ERX3333087	8	6	16	8
	ERX3333084	43	15	64	15
	ERX3333097	50	14	66	15
	ERX3333088	15	9	27	11
	ERX3333095	46	14	67	15
	ERX3333085	21	11	31	12
	ERX3333086	11	8	60	15
	ERX3333093	54	15	66	15
	ERX3333090	6	4	10	5
	ERX3333094	15	10	29	12
	ERX3333092	21	11	34	12
	ERX3333091	7	4	12	5
	ERX3333089	12	6	26	6
Metagenomics	SRX6447061	0	0	1	1
	SRX6447026	14	7	34	10
	SRX6447024	3	2	3	2
	SRX6447019	7	5	13	6
	SRX6447018	48	19	140	25
	SRX6446993	0	0	1	1

## Discussion

KARGAMobile is the first real-time Android app for detection of ARGs from Nanopore sequencing data, both whole genome as well as metagenomics, providing high accuracy and low false positive rates. KARGAMobile has a minimalist graphical interface and results are delivered, summarized in an interpretable way for the end user. The app works with off-the-shelf Android OS and its factory-set Java virtual machine, without any special configuration need.

One limitation of our tool is that it does not detect ARGVs; however, ARGV detection is a fundamentally different problem, since it involves the location and confirmation of specific point mutations, in addition to the identification of an ARG (Prosperi et al., 2019). Only a couple of tools are able to handle ARGVs, namely RGI and Pointfinder (Zankari et al., 2017; Alcock et al., 2019), but they process exclusively assembled genomes. A second limitation of KARGAMobile, as we mentioned in the methods, is that the implementation still make extensive use of raw String types, as well as HashMap data structures, which have a substantial memory padding. The overall RAM usage is contained through the compressed database, but discovery and addition of new genes in the future might require additional code optimization, or the usage of a disk-based hashing structure with minimal dependencies, e.g., mapDB (https://mapdb.org). A third limitation is that, although the device did not shut off for overheating in any of the trials, including the stress test made of 5-hour-long consecutive runs, the temperatures measured in our experiments were in the high range for a mobile device. Recent work showed that cache-oblivious *k*-mer data structures can decrease power dissipation more then 25% than non-cache-based (Milicchio and Prosperi, 2021). As a future perspective, we look forward to deploying an iPhone version of KARGAMobile.

In conclusion, KARGAMobile is a consumer-grade app that has broad employment potential, including stakeholders as public health officials, healthcare providers, and agricultural researchers.

## Data Availability

Publicly available datasets were analyzed in this study. This data can be found here: https://www.ncbi.nlm.nih.gov/sra/?term=PRJEB31907 and https://www.ncbi.nlm.nih.gov/sra/?term=PRJNA554461.
